# Nutraceuticals in HIV and COVID-19-Related Neurological Complications: Opportunity to Use Extracellular Vesicles as Drug Delivery Modality

**DOI:** 10.3390/biology11020177

**Published:** 2022-01-23

**Authors:** Sunitha Kodidela, Sandip Godse, Asit Kumar, Xuyen H. Nguyen, Alina Cernasev, Lina Zhou, Ajay Kumar Singh, Hari K. Bhat, Santosh Kumar

**Affiliations:** 1 Department of Pharmaceutical Sciences, University of Tennessee Health Science Center, 881 Madison Avenue, Memphis, TN 38163, USA; sgodse@uthsc.edu (S.G.); akumar23@uthsc.edu (A.K.); xnguyen3@uthsc.edu (X.H.N.); lzhou13@uthsc.edu (L.Z.); 2 Department of Clinical Pharmacy and Translational Science, University of Tennessee Health Sciences Center, Nashville, TN 37211, USA; acernase@uthsc.edu; 3 Department of Medicine, Division of Diabetes, Endocrinology, and Metabolism, Vanderbilt University Medical Center, 7465 Medical Research Building IV, 2215 Garland Avenue, Nashville, TN 37232, USA; ajay.singh@vumc.org; 4 Division of Pharmacology and Pharmaceutical Sciences, School of Pharmacy, University of Missouri-Kansas City, 2464 Charlotte Street, Kansas City, MO 64108, USA; bhath@umkc.edu

**Keywords:** nutraceuticals, dietary supplements, SARS-CoV-2, COVID-19, HIV, extracellular vesicles, drug delivery, central nervous system (CNS)

## Abstract

**Simple Summary:**

In this review, we discuss the potential use of extracellular vesicles (EVs) to deliver dietary supplements to the brain to reduce brain complications associated with HIV, COVID-19, and other brain disorders. Brain-related complications affect people with HIV and COVID-19 alike. Moreover, since HIV patients are at a higher risk of contracting COVID-19, their neurological problems can be exacerbated by COVID-19. The use of dietary supplements together with available treatment options has been shown to reduce the severity of infections. However, these treatments are not chemically compatible with the body’s blood–brain barrier defense mechanism. Therefore, a viable delivery method is needed to deliver drugs and nutraceuticals to the brain in HIV and COVID-19 comorbid patients.

**Abstract:**

People living with HIV/AIDS (PLWHA) are at an increased risk of severe and critical COVID-19 infection. There is a steady increase in neurological complications associated with COVID-19 infection, exacerbating HIV-associated neurocognitive disorders (HAND) in PLWHA. Nutraceuticals, such as phytochemicals from medicinal plants and dietary supplements, have been used as adjunct therapies for many disease conditions, including viral infections. Appropriate use of these adjunct therapies with antiviral proprieties may be beneficial in treating and/or prophylaxis of neurological complications associated with these co-infections. However, most of these nutraceuticals have poor bioavailability and cannot cross the blood–brain barrier (BBB). To overcome this challenge, extracellular vesicles (EVs), biological nanovesicles, can be used. Due to their intrinsic features of biocompatibility, stability, and their ability to cross BBB, as well as inherent homing capabilities, EVs hold immense promise for therapeutic drug delivery to the brain. Therefore, in this review, we summarize the potential role of different nutraceuticals in reducing HIV- and COVID-19-associated neurological complications and the use of EVs as nutraceutical/drug delivery vehicles to treat HIV, COVID-19, and other brain disorders.

## 1. Introduction

In 1981, a report on the diagnosis of five young men with pneumocystis carinii pneumonia signaled the start of the HIV pandemic [1]. Similarly, in late 2019, reports from Wuhan, China, about a new severe respiratory illness started the beginning of the COVID-19 pandemic [2,3]. Many mitigation methods or programs that were followed during the HIV pandemic were implemented during the COVID-19 pandemic to minimize the spread of infection [4]. The introduction of combination antiretroviral therapy (cART) has decreased the incidence and severity of HIV-associated neurocognitive disorders (HAND) and HIV-associated dementia (HAD) [5]. However, the prevalence of mild-to-moderate HAND is rising due to multiple factors, including poor permeability of anti-retroviral drugs (ARVs) across the blood–brain barrier (BBB) [5]. As observed in the case of HIV infection, there is a high probability that SARS-CoV-2 can enter the brain and cause neurological complications [6,7].

Based on the lessons learned from the previous HIV pandemic, one can expect that the neurological complications with COIVD-19 will continue to rise despite receiving the treatment for COVID-19. Recently, the number of COVID-19 patients experiencing neurological complications has been increased despite receiving the treatment for SARS-CoV-2 infection [8,9,10]. The co-infection of SARS-CoV-2 in people living with HIV/AIDS (PLWHA) [11,12] can further exacerbate the underlying neurological complication in PLWHA.

Initially, it was thought that PLWHA receiving ARVs, particularly protease inhibitors (PIs), would have less severe COVID-19 symptoms, as those drugs have shown activity against SARS-CoV-2. However, the evidence for this conclusion comes mainly from in vitro studies [13,14,15,16]. Furthermore, the antiretroviral drug remdesivir is the first FDA-approved drug for the treatment of COVID-19 [17]. Unfortunately, its use has declined as it was ineffective in reducing the mortality, initiation of ventilation, or hospitalization of patients with COVID-19 [18]. Similarly, PIs were also found to be ineffective in hospitalized adult patients with severe COVID-19 [18,19,20].

The risk of developing severe or fatal COVID-19 is 30% greater for PLWHA when compared to people without HIV infection [21]. Furthermore, PLWHA are at risk of COVID-19 despite treatment with ART [22,23]. Thus, the WHO urges PLWHA to receive COVID-19 vaccinations [21]. There is a social stigma surrounding the COVID-19 vaccine among some individuals and certain communities, although currently approved vaccines effectively reduce the spread of infection and hospitalization. Despite the reduction in risk of severe infection or hospitalization, many vaccine breakthrough cases have been reported [24,25]. This suggests that the virus is likely to continue to replicate, further increasing the likelihood of reaching the brain and causing neurological complications. Although a few reports have been published on patients experiencing neurological complications after receiving COVID-19 vaccinations [26,27,28], there is still a need to investigate the chronic effects of the COVID-19 vaccine on the immune system.

Plasmalogens, a class of membrane ether glycerophospholipids, play a role in immune signaling and as endogenous antioxidants [29]. Recently, decreased levels of plasmalogens were associated with reduced lung function [30] and disease progression [31] in COVID-19 patients. Therefore, plasmalogens have been suggested as anti-viral therapeutics and prophylaxis strategies to prevent coronavirus infections [32]. However, the research on plasmalogens in coronavirus infection is in its infancy, and further work in this area is necessary to explore the full potential of plasmalogens as therapeutic strategies in antiviral combination remedies.

While antivirals and vaccinations are in use to combat COVID-19, interestingly, many individuals have sought out non-pharmaceutical protection measures by consuming various dietary supplements and nutraceuticals that they believed to confer beneficial effects [33]. Nutraceuticals, such as a variety of phytochemicals from medicinal plants and dietary/herbal supplements, have been used as adjunct therapies for many disease conditions, including viral infections [34,35,36,37]. Derosa et al. have reported the role of nutraceuticals and botanical remedies as immunity enhancers in prophylaxis and cure of COVID-19 [38]. Further the immunomodulatory properties of nutraceuticals have been investigated by many researchers around the world on account of little proven therapeutic options in COVID-19 [39]. Flavonoid quercetin [40], myricetin, and scutellarein [41], which are found in many foods, have shown inhibitory activity against SARS-CoV-1. Most herbal nutraceuticals are widely studied for their anti-inflammatory activity, which can be exploited to control the cytokine storm associated with SARS-CoV-2 infection. Polyphenolic compounds such as curcumin display reasonable levels of inhibitory activity against IL-6, IL-1β, and TNF-alpha [42]. Some of the at-risk groups for severe illness associated with COVID-19 are older adults and people with underlying medical conditions such as cancer, chronic renal disease, pulmonary, neurological conditions, HIV infection, immunocompromised state, obesity, and pregnancy [43].

Although there is a lack of sufficient clinical evidence supporting the use of nutraceuticals and dietary/herbal supplements against COVID-19, many people have resorted to using herbal/dietary supplements and other nutraceuticals with the hope of improving their immune system and decreasing the risk of developing COVID-19. The global nutraceuticals and dietary supplements market has steadily grown over the past decade and was reported to be worth almost USD 353 billion in 2019 [39,44]. A PubMed search shows that between 2010 and 2021, over 70,000 manuscripts were published on dietary supplements and nutraceuticals.

Recently, an increasing number of in vivo, in vitro, and clinical studies have reported the emerging role of extracellular vesicles (EVs) in drug delivery and immunomodulation. The linkage between EVs and viral infection has led to exploring the possibilities for EVs as novel therapeutic carriers owing to the presence of the specific target molecule angiotensin converting enzyme 2 (ACE2) [45]. A recent study has reported that the incidence of mortality and severe morbidity associated with COVID-19 infection in HIV increases with the simultaneous presence of multimorbidity and old age [46]. EVs display a promising role as drug carriers with the current paucity of available treatment options in COVID-19.

Therefore, in this review, we discuss the neurological complications reported with HIV infection and SARS-CoV-2 infections. Then, we focus on the role of different nutraceuticals in treating neurological complications associated with HIV and COVID-19 infections. Furthermore, we discuss novel therapeutic delivery strategies using extracellular vesicles (EVs) to improve nutraceuticals’ delivery and their levels in the brain to reduce neurological complications associated with these viral infections.

## 2. HIV-Associated Neurological Complications

Since the introduction of cART, HIV infection has transformed from a deadly infection into a manageable chronic infection, allowing PLWHA to have normal to near-normal lives [47,48,49]. However, due to the limited amount of ART that can reach the brain, around half of HIV patients under treatment still experience HAND pathologies. The global prevalence of HAND is 42.6%, and it encompasses an array of neurocognitive dysfunctions associated with HIV infection, such as asymptomatic neurocognitive impairment (ANI), mild neurocognitive disorder (MND), and HAD [5,50]. HAND is diagnosed by functional status and neuropsychological testing [50]. It is important to note that although some PLWHA do not show symptoms, ANI may shift to one of the more severe forms of HAND [51,52]. A study showed that the PLWHA who experienced ANI at baseline were two to six times more likely to develop symptomatic HAND during several years of follow-up than those who were neurocognitively normal at baseline [52].

Patients with HAND present with various clinical manifestations, including memory impairment that manifests in attention disruption, challenges in multitasking, and judgment [51,53]. All these symptoms lead to executive dysfunction. As the disease advances, the individuals experience additional motor dysfunctions that present as bradykinesia, loss of coordination, and gait imbalance [51,53]. As the HAND progresses, the disease could result in dementia, and the person could be bed-ridden [53].

## 3. COVID-19-Associated Neurological Complications

**Figure 1:**
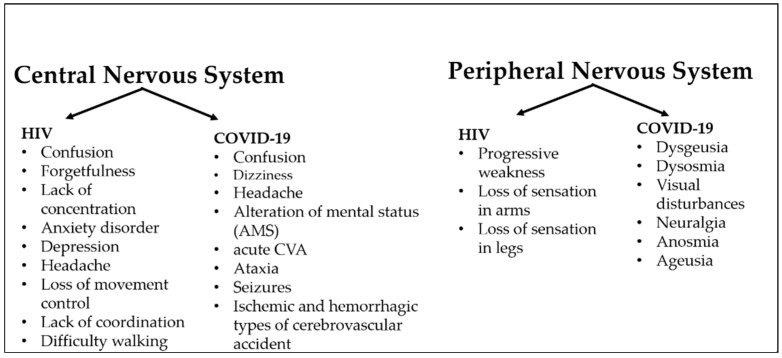
Major reported neurological complications associated with HIV and COVID-19.

Similar to people living with HIV, patients diagnosed with COVID-19 present with a variety of neurological symptoms [9,54]. The mechanism by which the virus enters the brain and produces diverse symptoms has not yet been fully elucidated. However, some reports suggest that SARS-CoV-2 utilizes angiotensin-converting enzyme 2 (ACE2) receptor to infect host cells [55,56,57]. ACE2 receptors are widely expressed in neurons, astrocytes, endothelium, and vascular smooth muscle cells [58,59]. A recent review assessed a total of 2533 hospitalized COVID-19 patients and reported that neurological symptoms were identified in 73% of the hospitalized patients [60]. The most commonly reported neurological symptoms in that study included headache, muscle pain, and loss of consciousness [60]. Another study investigated face-to-face encounters with physicians, and this cohort showed that 27.6% of participants presented with headache, 3.3% with trigeminal neuralgia, 3.7% with glossopharyngeal neuralgia, 3.8% had a cerebrovascular disease (CVD), and 15.1% presented with muscle pain [61]. Although most patients might experience some mild neurological symptoms, some studies reported worsening symptoms and the development of other symptoms such as seizures, anosmia, stroke, and impaired consciousness [62]. For instance, a case report described a young male whose symptoms worsened after experiencing headaches, generalized fatigue, and fever [63]. A case study reported that patients diagnosed with COVID-19 experienced motor-predominant peripheral nerve disorders. Furthermore, a female in the same case study also presented with neuropathy and myopathy [64]. The neurological complications associated with HIV and COVID-19 infection are summarized in Figure 1.

It has been observed that HIV and COVID-19 co-infected patients have a more severe clinical presentation than the general population [22]. However, currently, the research on COVID-19 infection in PLWHA is in its infancy, and future studies will provide more insights into the underlying mechanisms by which SARS-CoV-2 infection further aggravates the neurological complications in PLWHA.

The published studies suggest that the use of nutraceuticals may improve the immune system and decrease the likelihood of some diseases such as cold, flu, anxiety, depression, cardiovascular diseases, etc. [65,66,67,68,69]. The neurological complications can be exacerbated in co-infection of HIV and COVID-19 due to the ineffectiveness of ARTs. Therefore, it is possible that these people may try to mitigate their HIV- or COVID-19-related illnesses by resorting to dietary supplements/nutraceuticals. Thus, it is crucial to explore or review the literature concerning natural products that may help address this issue.

## 4. Role of Nutraceuticals/Dietary Agents in Alleviating Neurological Complications Associated with HIV and COVID-19

Table 1 describes the outcome of studies focusing on using nutraceuticals/dietary agents in treating HIV and COVID-19. We discuss the role of individual nutraceutical agents in neurological complications associated with HIV and COVID-19 below.

### 4.1. Vitamin D

Vitamin D is a secosteroid hormone, which is among the most important micronutrients that can serve as a modulator for both innate and adaptive immune responses [70,71]. An adequate level of vitamin D can be achieved through sun exposure, nutrition, or supplementation [71,72]. The vitamin D (cholecalciferol) undergoes hepatic metabolism and converts to 25-hydroxyvitamin D (25(OH)D) [70]. It is further converted into the most active metabolite 1, 25(OH)_2_D_3_ in the kidney [70]. 25-hydroxyvitamin D is considered a biomarker for vitamin D3 levels in the body [70]. The increase in serum 25(OH)D is associated with the decrease in risk and severity of SARS-CoV-2 infection through various mechanisms such as maintaining epithelial cell layers, increasing ACE2 receptor expression, and reducing the production of pro-inflammatory cytokines [70,71,72,73]. Additionally, vitamin D reduces the risk of infection by (1) enhancing the body’s physical barrier by regulating the production of proteins for tight junction, adherent junction, and gap junction and by (2) increasing production of antimicrobial peptides such as cathelicidin and human β defensins 2 [72]. A study in rats with acute respiratory distress syndrome (ARDS) demonstrated that the increase in serum 25(OH)D leads to an increase in levels of ACE2 mRNA and protein [70,71]. The increase in serum 25(OH)D also leads to the decrease in the production of proinflammatory cytokines such as TNF-α, IL-1β, IL-6, IL-12, INF-γ, and NF-κB, which helps prevent “cytokine storm” and other serious complications of SARS-CoV-2 infection [70,71]. In late 2020, the COVIT-TRIAL, a randomized controlled trial (RCT), investigated the effectiveness of vitamin D supplementation in COVID-19 patients. This trial concluded that a high dose of vitamin D could serve as an effective and safe adjunctive agent for the treatment of COVID-19 [71]. However, the excessive consumption of vitamin D leads to vitamin D toxicity, which is characterized by hypercalcemia, leading to serious health consequences [74]. Although more RCTs are still needed to provide more evidence on the effectiveness of high-dose vitamin D supplementation, it is generally recommended that a supplement dose of vitamin D (50,000 units of cholecalciferol per week for 3 months) can be given to high-risk populations and populations who have poor exposure to the sun [70,73].

There is no study suggesting the role of vitamin D in reducing HIV-associated neuronal damage or neurological complications. However, since HIV–COVID-19 comorbidity is a common occurrence, vitamin D is likely to reduce exacerbated neurological complications in these comorbid patients.

### 4.2. Vitamin C

Vitamin C (ascorbic acid) is an essential water-soluble nutrient that functions as a cofactor for numerous enzymatic reactions. Vitamin C also serves as an antioxidant, anti-inflammatory, immunomodulatory, anti-viral, and anti-thrombotic agent and can potentially be used as a therapeutic or prophylactic agent against SARS-CoV-2 [72,75,76,77,78]. The administration of vitamin C with methylene blue and N-acetylcysteine (an antioxidant) can improve the immune response in patients with COVID-19 by reducing serum nitrate (NO_3_^−^), methemoglobin, C-reactive protein, lactate dehydrogenase, and other inflammatory markers such as ferritin and D-dimer [72,77]. A high dose of vitamin C also inhibits glyceraldehyde-3-phosphate dehydrogenase (GAPDH), which may decrease the activation of immune cells and reduce inflammation [72]. Vitamin C is also an important antiviral agent due to its ability to promote lymphocyte activity, increase interferon-α production, improve endothelial and mitochondrial function, and support apoptosis and phagocytosis of neutrophils [78]. In fact, a study by Hernandez et al. suggests that the use of 30–60 g of vitamin C can effectively treat cytokine storm-generated increases in ROS, provided that the high levels of vitamin C do not promote enhanced chemotaxis of white blood cells [77]. The authors also suggested a protocol and precautions that should be taken into consideration to administer a high dose of vitamin C for treating COVID-19 patients [77]. Furthermore, a study by Biancatelli suggests that the concurrent use of quercetin (a plant flavonoid found in many common types of vegetables) with vitamin C may augment its antiviral and immunomodulatory effects. Thus, this combination shows promise as a potential prophylaxis and therapeutic treatment for SARS-CoV-2 [78].

Reduced levels of vitamin C have been found in patients with lung injuries, pneumonia, sepsis/septic shock, ARDS, and other critical illnesses [78]. Several trials (CITRIS-ALI, VICTAS, ACTS, HYVCTTSSS) are being conducted to investigate the ability of high-dose intravenous vitamin C (HDIVC) and oral vitamin C supplementations in alleviating these conditions [72,76,77,78]. When COVID-19 emerged, an RCT was conducted to investigate the correlation between administration of HDIVC and the improvement of SARS-CoV-2-induced ARDS [79]. However, the result of this trial shows no significant improvement in peripheral oxygen saturation and body temperature in the group that was treated with HDIVC in addition to the existing treatment [79].

Vitamin C acts as an active neuroprotector by removing free radicals in the brain and yields dehydroascorbic acid (DHA 5%) [80]. An in vivo study elucidated that DHA attenuates ischemic brain edema and suggested that vitamin C improves the cognitive decline in Alzheimer’s disease (AD) [81]. Oxidative stress plays an important role in the progression of HIV/AIDS by influencing viral replication, inflammatory responses, and decreased immune cell proliferation [82]. Patients with HIV have high oxidative stress and low vitamin C levels. However, there is no positive correlation between the supplementation of vitamin C and a reduction in oxidative stress [82,83]. It has also been suggested that daily use of 1000 mg of vitamin C for 7 days appears to induce certain hepatic cytochromes, including cytochrome P450 3As (CYP3As), which leads to a decrease in the Cmax of indinavir by 20% [84]. More studies and trials are needed to draw a conclusion on the benefits of vitamin C on disease progression and prognosis of HIV infection.

### 4.3. Ginkgo Biloba (GB)

Ginkgo biloba contains quercetin, a polyphenol compound, and other components, and is suggested to have antiviral, antioxidant, anti-inflammatory, and immunomodulatory effects that decrease pro-inflammatory cytokines [85]. Ginkgo biloba contains two different groups of compounds: (1) flavonoid glycosides (rutin and quercetin) and (2) terpene lactones (ginkgolic acid and ginkgolides A–C) [86]. Ginkgolic acid (GA) produced by GB can act as an inhibitor against HIV and other enveloped viruses through the inhibition of protease activity [86,87]. GA is also widely used as a natural therapeutic agent to improve memory and treat conditions such as hemorrhoids, dementia, and depression [84].

Experiments in HIV trans-activator of transcription (Tat) transgenic mice indicate that EGb 761 (a standardized formulation of GA extract) exhibits neuroprotective properties by down-regulating the expression of GFAP (Glial Fibrillary Acidic Proteins) in the brain, which negates the astrocytosis that occurs in response to HIV-related brain injuries. EGb 761 also directly and negatively affects the binding of AP-1 and NF-κB transcription factors on the GFAP promoter, which conceivably results in a decrease in the infiltration of pro-inflammatory cytokines into the brain [88]. Considering the unwanted side effects and increasing resistance to traditional protease inhibitors (PIs), GA can serve as a natural, effective, and less aggressive alternative. Lu et al. reported that GA inhibits HIV protease activity in a concentration-dependent manner in a cell-free system (by 60% compared to placebo) and in HIV1SF162-infected human peripheral blood mononuclear cells (PBMCs) [86]. Mango et al. [89] demonstrated that GA exhibits neuro-inhibitory effects against amyloid-β (Aβ)-induced impairment of synaptic plasticity and neurotransmitter release [89,90]. GA, perhaps via the Bcl-2/Bax pathway, contributes to anti-viral and Aβ-mediated impairment [89,90]. Published studies also indicate that the dysregulation of the PI3K/Akt/mTOR pathway by GA might be an inhibitory mechanism against Aβ [89]. However, several studies have shown that the terpene-containing GA may induce CYP3A4 and P-gp, leading to an increase in viral loads from undetectable to detectable levels of 1350 copies/mL in HIV/AIDS patients who are treated with efavirenz. Thus, drug interactions must be taken into consideration, and the concurrent use of GA and efavirenz is generally not recommended [84].

A recent review by Ibrahim et al. suggests that quercetin, a flavone extract of GB, can serve as an anti-inflammatory and immunomodulatory agent by the downregulation of many pro-inflammatory cytokines such as lipopolysaccharide (LPS)-induced tumor necrosis factor α (TNF-α), LPS-induced IL-8 in A549 cells in the lungs, and LPS-induced IL-1α and TNF-α in glial cells [85]. GA, an alkylphenol constituent of GB, has been shown to inhibit the viral protein-mediated host cell fusion in a variety of enveloped viruses (EBOV, HIV, ZIKA, HSV-1, HCMV, EBV, and IAV) and some non-enveloped viruses, which suggests that GA can potentially be used to treat other acute infections, including SARS-CoV-2 [85,87]. A recent study by Xiong et al. also found that three ginkgo biloba leaf extracts (GBLEs)—GA C15:0, GA C17:1, and the bioflavone sciadopitysin—inhibit 3-Chymotrypsin-like protease (3CLpro), a virally encoded proteinase essential for the replication of SARS-CoV-2, in a reversible and mixed-inhibition manner [91]. This finding holds promise for the development of novel treatments for SARS-CoV-2 utilizing these GBLEs.

### 4.4. Green Tea

Tea is the most widely consumed beverage [92,93]. Green tea, black tea, and white tea are extracted from the plant camellia sinensis [94]. Among the most common types of tea, green tea accounts for approximately 20% of manufactured tea. Polyphenols are the major components of green tea, and major polyphenols are flavonoids. Among the four types of flavonoids, epigallocatechin-3-gallate (EGCG) is considered the most active [93]. EGCG is found to be a potent inhibitor of influenza virus replication, the effects of which were observed in several subtypes such as A/H1N1, A/H3N2, and B [95]. EGCG also exhibits antiviral effects against HIV via several potential mechanisms [93].

In patients with HAD, neuronal damage and apoptosis are triggered by HIV proteins, Tat and envelope glycoprotein 120 (gp120), and the activation of the Janus-associated kinases/signal transducer and activator of the transcription (JAK/STAT1) pathway [96,97]. In vitro and in vivo experiments on HIV Tat transgenic mice suggest that EGCG derived from green tea flavonoids helps impair the STAT1 pathway, thus attenuating the IFN-γ-mediated JAK/STAT1 pathway, and can potentially be used as adjunctive components to cART [96,98]. A study by Nath et al. suggested that in Tat transgenic mice brains, there is a decrease in the mature brain-derived neurotrophic factor (BDNF) signaling pathway and an increase in proapoptotic pro-BDNF, which induces neuronal apoptosis [99]. Thus, by targeting pro-BDNF and BDNF, as well as preventing the formation of reactive oxygen species (ROS), EGCG may serve as a potent neuroprotective and antioxidative agent [99]. Moreover, EGCG can be used as a therapeutic agent against AD due to its ability to enhance α-secretase and reduce β- and γ-secretase. EGCG can also inhibit the deposition of β amyloid peptide in vitro with a maximum inhibitory concentration of 7.5 mg/L [100].

In a more recent study, Smith et al. attempted to encapsulate ECGC in nanolipidic particles, which improved EGCG’s α-secretase-enhancing ability by 91% in vitro and oral bioavailability twofold in vivo, compared to free EGCG [101]. This suggests potential benefits in nanoparticle encapsulation and surface modifications of ECGC molecules. Furthermore, EGCG can potentially serve as an anti-inflammatory and anti-fibrosis agent against viral infection by SARS-CoV-2, which stems from its ability to upregulate neprilysin (NEP) expression in the lungs [102].

### 4.5. Resveratrol

Resveratrol (trans-3,5,4′-trihydroxystilbene) is a natural polyphenol that is found in peanuts, berries, grapes, and red wine and can serve as an anti-inflammatory, anti-viral, and anti-aging agent [103,104,105]. In an ex-vivo experiment of hippocampal slices of HIV Tat transgenic mice, Lee et al. demonstrated that resveratrol significantly reduces the extracellular signal-regulated kinase (ERK) 1/2 activation and Tat-mediated production of TNF-α, thereby downregulating the release of monocyte chemotactic protein 1 (MCP-1/CCL2). This suggests a possible anti-inflammatory and neuroprotective mechanism exerted by resveratrol to the Tat-exposed hippocampus [103]. Sirtuins (1-7) (SIRT 1-7) induced by resveratrol is a class of deacetylase enzymes linked to metabolic control, gene expression, aging, and neuroprotection. By activating SIRT1, resveratrol can be useful in the treatment and prevention of neuronal loss [106].

The activation of SIRT1 and p53-mediated apoptosis are also the key mechanisms for resveratrol antiviral activity against influenza virus, MERS-CoV, and SARS-CoV-2 [107]. Recent studies suggest that resveratrol modulates host immune response by inhibiting pro-inflammatory cytokines (IFN-γ, TNF-α, IL-1β) and activating cytotoxic T lymphocyte (CTLs) and NK cells, thus mitigating lung damage upon SARS-CoV-2 infection [105,107]. Using molecular dynamics simulation and free binding energy analysis, Wahedi et al. found that resveratrol binds to ACE2 with high binding energy, forming a stable complex, which suggests the use of resveratrol as a disruptor at the SARS-CoV-2 spike protein and ACE2 binding interface [108]. However, due to its complex structure, hydrophilicity, and rapid metabolism, resveratrol has low bioavailability and is less effective in crossing the BBB [99,105]. To combat this issue, studies have investigated the synthesis of novel resveratrol analogs such as methoxylated, hydroxylated, and halogenated resveratrol, showing enhanced lipophilicity and bioavailability in vitro. Some of the resveratrol analogs have shown better antioxidant activities than the parent compound [109,110]. Abdalla et al. reported that one resveratrol analog 4-(E)-{(p-tolylimino)-methylbenzene-1,2-diol} (TIMBD) exerts anti-inflammatory effects better than that of resveratrol in SVG astrocytes in vitro [111]. TIMBD has been shown to suppress HIV1-gp120-mediated production of IL6 and IL8 [111]. TIMBD also reduced gp120-mediated phosphorylation of cJUN, cFOS, STAT3, p-38-MAPK, AKT, and IKKs [111]. Furthermore, TIMBD decreased nuclear translocation of the NF-kB p-65 subunit, whereas resveratrol mostly affected the protein expression levels of NF-kB [111]. These results suggest that this resveratrol analog may have the potential of being a novel agent for treating HIV1-gp120-mediated neuroinflammatory diseases [111]. A more promising method is nanoparticle encapsulation, which has shown improved chemical stability, BBB penetration, and fewer side effects both in vitro and in vivo [105].

### 4.6. Curcumin

Curcumin is a hydrophobic polyphenol that can be derived from the plant turmeric. In recent years, curcumin has been widely explored for its anti-oxidative, anti-inflammatory, anti-tumor, and neuroprotective properties [97,112,113,114,115,116,117,118]. After treating HIV gp120-infected primary rat cortical neurons with curcumin, Guo et al. found that curcumin helps impair neuronal apoptosis by reducing gp120-mediated production of ROS and other pro-inflammatory cytokines such as TNF-α, IL-1β, and MCP-1 [97]. In a similar study, Xia et al. found that curcumin reduces neuronal apoptosis by increasing the expression of 70 kDa heat shock protein (HSP70) in a dose-dependent manner [112]. Boris and colleagues demonstrated that HSP70 inhibited neuronal apoptosis by modulating both caspase-dependent and caspase-independent pathways [119]. Furthermore, the neuroprotective activity of HPS70 may be due to its role as a chaperone that reduces protein aggregation and toxicity. In addition, HSP70 exerts neuroprotective activity due to its direct anti-apoptotic activity [120]. Curcumin can also produce an effect similar to nimodipine, which helps improve synaptic growth, relieve intracellular calcium overload, and inhibit L-type calcium current in the rat gp120 hippocampal neurons [121,122]. A recent study by Zhao et al. also found that encapsulating curcumin in biodegradable nanoparticles not only improves the bioavailability and BBB penetration of curcumin but also reduces the expression of P2*X*_3_ receptor in the dorsal root ganglia (DRG), which helps alleviate neuropathic pain in gp120 rats brain cells [113]. Furthermore, in vitro and animal studies also suggest that curcumin is effective at slowing down the progression of AD by interfering with β amyloid metabolism [117].

A recent review by Thimmulappa suggests the use of curcumin as a prophylactic and therapeutic agent against SARS-CoV-2 infection due to its relatively large safety profile on human subjects, broad antiviral spectrum against many types of enveloped viruses including SARS-CoV-2, and immunomodulatory activity. Curcumin’s ability to downregulate proinflammatory cytokines such as NF-κB, inflammasome, HMGB1, and IL-6 helps prevent the “cytokine storm” that eventually leads to ARDS and septic shock in severely ill COVID-19 patients [123]. SARS-CoV-2 infection is initiated by the binding of the viral S glycoprotein to its ACE2 receptor in alveolar cells [115,116,124,125]. Several studies have targeted this pathway in the potential treatment and prevention of SARS-CoV-2 infection. Recent molecular docking studies found that curcumin can bind to both subunit S1 of S glycoprotein and ACE2 with high affinity, thus inhibiting the viral binding [108,115,116,125]. Once the spike protein binds to ACE2, its subunit S2 is primed and cleaved by protease serine 2 (TMPRSS-2) and protease cathepsin B and L (Cat B/L) [116,125]. By diminishing the activity of TMPRSS-2 and Cat B/L, curcumin can inhibit the release of viral content into cells [116]. Furthermore, curcumin can also suppress S glycoprotein replication, with an EC50 value of higher than 10 µM [115]. Therefore, curcumin may prove to be a promising natural remedy for HAD and SARS-CoV-2 infection. Recent studies have suggested nano formulation using carriers such as liposomes, niosomes, lipid complexes, micro/nano emulsions, and polymeric nanoparticles can significantly improve pulmonary delivery of curcumin to the lower airways and alveolar region at high concentrations [123].

**Table 1:** Outcome of studies focusing on using nutraceuticals/dietary agents in treating HIV and COVID-19.

Drug	HIV Outcomes	COVID-19 Outcomes	References
Ginkgo biloba (GB) and related compounds	↑ HIV protease activity	---	[86]
	Show neuroprotective effects	---	[88]
	---	↓ enveloped viral fusion Show antiviral activity against influenza virus	[85,87]
	---	Inhibit vital proteinase SARS-CoV-2 3CL^pro^	[91]
Vitamin D supplementation	↑ 25(OH)D concentration, correct vitamin D deficiency, improve immune response, and reduce mortality in HIV-positive patients	---	[126,127,128]
	Improve maternal health, birth outcomes, and infant growth among HIV-infected pregnant women	---	[129]
	---	Enhance the immune system, reduce risk, severity, and improve prognosis of SARS-CoV-2 infection	[71,72,73]
	---	Prevent “cytokine storm”	[130,131,132,133,134]
Vitamin C (ascorbic acid)	↓ neuronal damage, but ↑ increase risks of drug interactions when used with multiple ARVs	---	[81,84]
	---	Enhance immune system and reduce severity of SARS-CoV-2 infection	[72]
	---	Intravenous (I.V.) vitamin C can be an effective treatment	[77]
	---	Can be used concurrently with Quercetin as a prophylactic agent and treatment option	[78]
	---	HDIVC provides no significant outcome in nCoV-2-induced ARDS	[79]
Green tea (and other EGCG-containing compounds)	Antiviral effect against influenza, HIV, and hep C	---	[93]
	↓ neuronal damage and apoptosis	---	[96,98]
	↓ neurotoxic effect of HIV proteins and can cross BBB	---	[99]
	EGCG-containing nano-lipidic complexes enhance EGCG bioavailability by 2x	---	[101]
	↓ Aβ accumulation	---	[135]
Resveratrol	↓ the HIV viral replication caused by Benzo(a)pyrene	---	[36]
	↓ neurotoxic effects of HIV proteins	---	[99]
	Inactivate ERK1/2 pathways, which reduces TNF-α and MCP-1 production in the hippocampus	---	[103]
	Activate SIRT-1 in vivo by increasing NAD+ expression	---	[106]
	---	Show strong binding affinity to ACE2 in the lungs	[108]
Resveratrol analog- 4-(E)-{(p-tolylimino)-methylbenzene-1,2-diol} (TIMBD)	↓ HIV-gp120-induced neuroinflammation in SVG astrocytes	--	[111]
Curcumin	↓ ROS and proinflammatory cytokines	---	[97]
	↓ neuronal apoptosis, especially through the HSP70 pathway	---	[97,112]
	↓ neuropathic pain	---	[113]
	↓ Ca^2+^ concentration in synaptosomes	---	[121,122]
	↓ viral replication exacerbated by extracellular vesicles (EVs) derived from cervical cancer cell lines (CASKI)	---	[136]
	---	Anti-inflammatory and pulmonary-protective effects on the infected lung tissues	[115]
	---	↓ interactions of SARS-CoV-2 spike proteins to ACE2 receptors in lungs	[124]
	---	↓ severity of SARS-CoV-2 infection	[116,125]

**HDIVC**: High-dose intravenous vitamin C; **EGCG**: (-) epigallocatechin—3-gallate; **GFAP**: glial fibrillary acidic protein; **NAD+**: nicotinamide adenine dinucleotide; **HSP70**: heat shock protein 70; **DRG**: dorsal root ganglia; **SIRT-1**: sirtuins 1; **GBLE**: gingko biloba leaves extract; **SVCT2**: Na/vitamin C co-transporter isoform 2. ↑ increased; ↓ decreased.

## 5. Extracellular Vesicles (EV)-Loaded Nutraceutical Agents for the Treatment of Neurological Complications Associated with HIV and COVID-19

With the unique ability to carry biomolecules, EVs have been attracting global attention to explore their therapeutic use against a variety of diseases, including central nervous system (CNS) and non-CNS diseases. EVs are broadly classified based on their size and origin pathway. For instance, exosomes, ranging from 30 to 150 nm [137], originate from inward budding of the membrane of early endosomes that eventually mature into multivesicular bodies and release into the extracellular space to mediate cell–cell communication [138]. On the other hand, microvesicles, ranging from 50 to 1000 nm, are produced through outward budding of cellular membrane. Apoptotic bodies, up to 5000 nm in size, are mainly produced by outward bulging of plasma membrane of apoptotic cells [139,140]. Researchers use EVs to load drugs and biomolecules and direct them to the infected or target cells to treat various diseases [137,138]. To consider EVs as personalized therapeutic carriers, surface modification of EVs is required [141,142,143,144]. However, it is highly desirable to optimize the method starting from cell selection to isolate EVs to the effective delivery route.

### 5.1. EVs as Drug Delivery System

With the recent advancements in drug delivery research, EVs have been explored for their role as an effective drug delivery system to deliver drugs and other molecules across the BBB [145,146]. EVs can be delivered with or without modification, and carry the therapeutic molecules to the targeted sites with less/no immunogenicity and toxicity [147]. EVs are biological nanoparticles that play a potent role in intercellular communication by carrying lipids, proteins, and coding and noncoding RNAs [148,149]. In addition to their physiological role, recent research demonstrated the pathological role of EVs involving the progression of diseases [150].

EVs can carry molecular cargo and contribute to many biological processes, including apoptosis [151]. With this unique ability, they are potential candidates for delivering therapeutic molecules to the target cells and tissues, including the brain and its residing cells. EVs in their pathological role may alter or contribute to disease state [152,153,154,155]. In addition to EVs, synthetic nanoparticles or biomaterials have been subjected to various research investigations to pursue their role in drug delivery in several diseases [137,156,157,158]. Different biomaterials have been investigated for therapeutic use in different disease models. Some are preferably used as therapeutic entities while others are found to be suitable candidates for drug delivery as carriers. Micelles have been formulated with curcumin [159] for therapeutic purpose and have been tried in several neurodegenerative diseases, including Alzheimer’s disease (AD), Parkinson’s disease (PD), and multiple sclerosis (MS). However, the implications of synthetic nanoparticles in clinics are limited due several reasons, one being their biodegradability [160,161,162]. Hence, the development of physiological nanoparticles is warranted so that they can effectively carry drugs despite their hydrophilic and hydrophobic nature and ensure drug delivery to target cells. The natural nanoparticles should also have the ability to cross to the BBB in order to provide therapy for neurodegenerative diseases, and then degraded without causing any harmful effects to the CNS. Being physiological nanoparticles, EVs provide an advantage over synthetic nanoparticles. EVs have been investigated for their immunogenicity, toxicity to cells, and tissue-penetrating ability, including brain tissue. Naturally occurring EVs attract a large number of researchers across the globe due to numerous advantages over biomaterials in context of a safe and effective drug delivery approach, especially for the therapy of neurodegenerative diseases [163]. EVs may have sufficient drug loading efficiency and loading capacity compared to other nanoparticles with low/no toxicity [146,164]. Being a natural carrier, EVs can effectively carry biomolecules such as lipids, proteins [165], and coding and non-coding RNAs that can regulate gene expression in the target cells, and thus provide suitability as an effective drug delivery system for biological molecules [138]. Moreover, EVs’ surface can be modified for better targetability to the desired cells, tissues, and organs. EVs also have the ability to cross the BBB naturally and transmit its cargo to the brain cells. However, to enhance the bioavailability of EV-encapsulated drugs, special steps must be taken for the successful integration of drugs into EVs, such as consideration of the nature of the drugs being loaded. Furthermore, before using EVs in clinics, the limitations of EVs should be addressed, such as stability, the nature of drugs, EV–drug pharmacokinetics, drug targets, drug metabolism, and immune clearance, in addition to sterile EV drug preparations on a large scale.

#### Different Strategies/Techniques for Loading Cargos in EVs

Multiple techniques have been developed to ensure cargo is loaded efficaciously into EVs, as this is an essential qualification to guarantee that EVs can sustain cargo for delivery. Approaches utilized for cargo loading are categorized as exogenous (use of physical treatment after EV isolation) and endogenous (incubating cargos with exosomes). The endogenous approach involves co-incubation, which exploits the sorting machinery of cells to produce EVs loaded with cargo. Although this loading technique is simple and gentle, retaining EV membrane integrity, it results in inefficient loading and thus limits its wide application. The low loading efficiency of the co-incubation failure can be attributed to the limited gradient-based diffusion and impervious EV membranes, which restrict easy access for hydrophilic cargo.

An alternate approach to exogenous loading involves the application of physical treatments, e.g., electroporation, ultrasound, saponin permeabilization, freeze–thaw cycle, and sonication. These methods create micropores in EV membranes, allowing rapid access for cargo and thus significantly improving loading. The approach of the physical treatment method is not without limitations; it is pertinent to carefully monitor damaged EV membranes and inactivation of cargo to achieve optimum loading efficiency with minimal damage.

Clinical translation of EVs remains challenging, despite the ideal characteristics of EVs as nanocarriers. The major hurdle is the lack of a promising production method, which assures better yield with the finest quality. Currently, most of the EVs produced in laboratory settings are obtained from human cells cultured in T-flasks that are subsequently ultra-centrifuged [166]. Under these laboratory conditions, EV yield is approximately 10^9^–10^11^ EVs per liter of culture media, which is hardly enough to test in mice [167]. The most common purification method used in laboratories is ult racentrifugation, an application which faces serious large-scale challenges due to the need for heavy rotors, multiple dead times due to batch processes resulting in reduced productivity, and damaged EV structure under high shear force [168,169,170]. A Good Manufacturing Practice (GMP)-grade EV production method produces high yield and high-quality EV therapeutic cargo, thus providing an adequate amount to use in preclinical and clinical settings with persistent output. A summary of different cargo loading techniques has been presented in Table 2.

### 5.2. EVs/Exosomes-Loaded Nutraceutical Agents-Based Therapies for HIV and Other CNS Disorders

EVs derived from cells can incorporate cellular proteins [200], lipids, and coding and non-coding RNAs to mediate intercellular communication. The delivery of EV-encapsulated drugs could affect target cells by altering signaling pathways. Moreover, incorporating drugs can trigger anti-inflammatory, anti-viral, anti-proliferative, and cytotoxic effects in target cells (Table 3).

The administration of EV/exosomal curcumin (Exo-Cur) in an LPS mouse septic shock model decreased inflammation in the lungs of mice [175]. Exo-Cur was also efficacious in multiple cellular and mouse models in which it directly affected CNS cells [201,202,203,204]. For example, it reduced microglial activation in the LPS-induced brain inflammation model [204] and delayed brain tumor growth in a GL26 tumor model [204]. Furthermore, curcumin loaded in engineered exosomes (expressing scFv of a high-affinity HIV-specific monoclonal antibody, 10E8, on exosome surface) specifically targeted cells expressing HIV viral envelop proteins and induced cell death, suggesting an approach to target HIV-infected in cells in brain reservoirs [201]. These findings were also validated in an NCG mouse model grafted with tumorigenic Env^+^ CHO cells [201]. In another study, the administration of Exo-Cur in rats was shown to reduce oxidative stress and decrease neuronal apoptosis in an cerebral ischemia–reperfusion injury model [202], and similarly showed a reduction in neuronal cell death in an AD mouse model [203].

In addition to curcumin, paclitaxel and other nutraceuticals have also been loaded into EVS and their effects in various cancer cell lines and mouse models have been studied [171,205,206,207]. Exosomal paclitaxel (Exo-Paclitaxel) was shown to induce cytotoxicity in multi-drug-resistant cancer cells [171] and it reduced pulmonary metastases in a mouse model of murine Lewis lung carcinoma pulmonary metastases [171]. Moreover, Exo-Paclitaxel combined with exosome anthocyanidins inhibited significant tumor growth in an ovarian cancer (A2780) tumor xenograft mouse model [205]. Another nutraceutical, berry anthocyanidins, was loaded in exosomes and studied in various cancer cell lines, showing anti-proliferative and anti-inflammatory activities [206] and reducing tumor growth in a mouse model [205]. An EV–black bean phytochemical formulation showed anti-proliferative activity in MCF7, Caco2, PC3, and HepG2 cancer cell lines [207]. Likewise, an exosome celastrol formulation exhibited enhanced anti-tumor efficacy in a human A549 lung cancer xenograft mouse model [208].

## 6. Conclusions

The prevalence of HAND and the challenge of therapeutics to cross the BBB are well known in literature. This is further complicated in PLWHA who contract SARS-CoV-2, as it also causes neurological complications. Currently, there are no established treatment options for SARS-CoV-2 and its causal neurological complications. The evidence for using nutraceuticals to mitigate neurological complications associated with HIV and SARS-CoV-2 comes from in vitro and in vivo studies. Hence, randomized clinical trials should be conducted to establish the clinical utility of nutraceuticals in HIV and SARS-CoV-2 infection-associated complications. Although these nutraceuticals relieve peripheral symptoms associated with HIV and SARS-CoV-2 infections, they may not alleviate neurological complications associated with these infections due to their inability to cross theBBB. EVs, as a drug carrier modality, have improved outcomes in various CNS disorders. Therefore, EV-loaded nutraceuticals have potential to improve delivery to the brain and thus improve the neurological outcomes in patients infected with both HIV and SARS-CoV-2.

## Figures and Tables

**Table 2:** A summary of different cargo loading techniques.

Method/ Technique	Type of Cargo	Advantages	Limitations	Cargo/ Drug	Disease	References
Coincubation	Hydrophobic	Simple technique	Low encapsulation efficiency	siRNAs	Neurodegenerative disorders	[171,172,173]
				miRNA-145	Cancer	[174]
				siRNAs	Huntington’s disease	[173]
				Curcumin	Inflammatory disorders	[175,176]
				Doxorubicin	Breast cancer	[177]
				Aspirin	Breast and colorectal cancer	[178]
				Gemcitabine	Pancreatic cancer	[179]
				Paclitaxel and doxorubicin	Brain cancer	[180]
Transfection	Hydrophilic/Hydrophobic	Improved molecular stability Improved chemosensitivity	Transfection agents may alter gene expression inducing anomalous biological activities	miR-128-3p	Cancer	[181]
				miR-146b	Glioma	[182]
				miR-143	Prostate cancer	[183]
				miR-let-7a	Breast cancer	[184]
				siRNA	Chronic myeloid leukemia	[185]
				miR-497	Lung cancer	[186]
Electroporation	Large molecules	Relatively simple High efficiency	Cargos form aggregates	Paclitaxel	Cancer	[171]
				miRNAs	Hepatocellular carcinoma	[187]
				siRNA	Pancreatic cancer	[188]
				Doxorubicin	Breast, ovarian cancer	[189]
				mRNA	Glioma	[190]
Ultrasound	Hydrophobic	High efficiency	Disrupts membrane, resulting in loss of integrity and stability	CTG	Cancer	[191]
				BSA FITC	Cancer	[191]
Saponin permeabilization	Protein	High efficiency	Residual saponins in membrane disrupts membrane integrity	Catalase	Parkinson’s disease	[192]
				DNA-oligonucleotide		[193]
				TPP1	Batten disease	[194]
				Porphyrins	cancer	[195]
Freeze–thaw cycle	Protein	Moderate efficiency	Freeze–thaw cycle disrupts membrane	Neprilysin	Alzheimer’s Disease	[196]
				hCG	Infertility	[197]
				Catalase	Parkinson’s disease	[192]
Sonication	Large molecules	High efficiency Sustained cargo release	Destroys membrane integrity and stability Exosomal aggregation	Paclitaxel	Cancer	[171]
				siRNA	Breast cancer	[198]
				Catalase	Parkinson’s disease	[192]
				Paclitaxel and Doxorubicin	Breast cancer	[199]
				Gemcitabine	Pancreatic cancer	[179]

**Table 3:** EVs/exosomes loaded nutraceutical agents-based therapies for HIV and other CNS disorders.

EV/Exosome Loaded Drug	Targeted Disease/Cells	Route of Administration	Mechanism	Reference
Exo-Curcumin	CHO cells expressing a trimeric gp140 on their surface (Env^+^ cells)	In vitro	↑ HIV-infected cell death	[201]
	NCG mouse model grafted with tumorigenic Env^+^ CHO cells	I.V. injection	↓ strong suppression of the Env^+^ tumor growth with low toxicity	
	Cerebral ischemia–reperfusion injury (rats)	I.V. injection	↓ ROS accumulation in ischemic lesions, alleviated BBB damage and suppressed mitochondria-mediated neuronal apoptosis.	[202]
	Alzheimer’s disease (mice)	I.P. injection	↓ okadaic acid induced neuronal cell death by ↓ hyperphosphorylation of Tau protein through the AKT/GSK-3β pathway	[203]
	LPS-induced brain inflammation model; EAE mice; GL26 brain tumor mouse model	Intranasal	↓ microglia activation; ↓ IL-1b expression in CD45.2 microglial cells; ↓ brain tumor growth	[204]
**Non-CNS diseases**				
Exo-curcumin	LPS mouse septic shock model	I.P. injection	↓ CD11b+Gr-1+ cells in the lungs of mice; anti-inflammatory	[175]
Exo-paclitaxel (alkaloids)	Human ovarian cancer A2780 cells xenograft in female athymic nude mice	Oral delivery	↓ significant tumor growth	[205]
	Multi-drug-resistant cancer cells (3LL-M27, MDCK wt, MDCK MDR1)	In vitro	↑ cytotoxicity	[171]
	Mouse model of murine Lewis lung carcinoma pulmonary metastases	Intranasal	↓ pulmonary metastases growth	[171]
	Ovarian cancer OVCA433 cells	In vitro	Anti-proliferative activity	[205]
	Human ovarian cancer A2780 cells xenograft in female athymic nude mice	Oral gavage	↓ tumor growth	[205]
Exo-berry anthocyanidins	Cancer cell lines (lung cancer: A549, H1299; breast: MCF7, MDA-MB-231; colon: HCT116; pancreatic: Panc1, Mia PaCa2; prostate: DU145, PC3; ovarian: Ovca432)	In vitro	Antiproliferative and anti-inflammatory effects in vitro	[206]
	Athymic nude mice bearing subcutaneous lung cancer A549 xenografts	Oral gavage	↑ therapeutic response of it against lung cancer tumor xenograft	[206]
EV–black bean phytochemicals	MCF7, caco-2, PC3, and HepG2 cancer cell lines	In vitro	Antiproliferative activity	[207]
Exo-celastrol	Human A549 lung cancer xenograft mouse model	Oral gavage	↑ anti-tumor efficacy	[208]

## Data Availability

No new data were created or analyzed in this study. Data sharing is not applicable to this article.

## List of Abbreviations

ACE2	Angiotensin-converting Enzyme 2
AD	Alzheimer’s disease
ANI	Asymptomatic neurocognitive impairment
ARDS	Acute respiratory distress syndrome
ARVs	Antiretroviral drugs
Aβ	Amyloid-β
BBB	Blood–brain barrier
BDNF	Brain-derived neurotrophic factor
cART	Combination antiretroviral therapy
CNS	Central nervous system
COVID-19	Coronavirus disease 2019
CVD	Cardiovascular disease
DHA	Dehydroascorbic acid
EGCG	Epigallocatechin-3-gallate
EVs	Extracellular vesicles
Exo-Cur	Exosomal curcumin
Exo-Paclitaxel	Exosomal paclitaxel
GA	Ginkgolic acid
GBLEs	Ginkgo biloba leaf extracts
HAD	HIV-associated dementia
HAND	HIV-associated neurocognitive disorders
HDIVC	High-dose intravenous vitamin
I.P.	Intraperitoneal
I.V.	Intravenous
LPS	Lipopolysaccharide
MS	Multiple sclerosis
PD	Parkinson’s disease
PIs	Protease inhibitors
PLWHA	People living with HIV/AIDS
RCT	Randomized controlled trial
SARS-CoV-2	Severe acute respiratory syndrome coronavirus 2
TIMBD	4-(E)-{(p-tolylimino)-methylbenzene-1,2-diol}
